# 1,1′-Methyl­enebis{4-[(*E*)-2-(pyridin-4-yl)ethen­yl]pyridinium} dibromide dihydrate

**DOI:** 10.1107/S2414314622005259

**Published:** 2022-05-20

**Authors:** Henry C. Neal, Volodymyr V. Nesterov, Bradley W. Smucker

**Affiliations:** a Austin College, 900 N Grand, Sherman, TX 75090, USA; b Department of Chemistry, University of North Texas, 1508 W. Mulberry, Denton, TX, 76201, USA; University of Antofagasta, Chile

**Keywords:** crystal structure, pyridinium, hydrogen bonding, π–π inter­actions

## Abstract

The chevron-shaped cations of the title hydrated salt, C_25_H_22_N_4_^2+^·2Br^−^·2H_2_O, are arranged in back-to-back alternating directions to form a zigzag ribbon propagating along the [010] direction. Inter­molecular inter­actions comprising these ribbons are π–π inter­actions between the pyridinium and adjacent pyridyl rings, as well as O—H⋯O hydrogen bonding between water molecules and two adjacent pyridyl N atoms. Half of the cation is generated by the mirror plane. The water O atoms, the central C atom and one Br atom are located on this mirror plane while the other Br atom is on a twofold screw axis.

## Abstract 2

Cations of the title compound inter­act *via* π–π inter­actions and hydrogen bond with water to form a zigzag ribbon.

## Graphical abstract

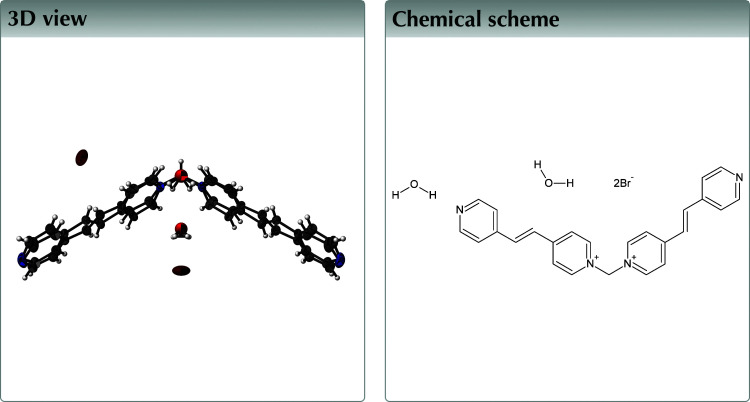

## Structure description

**Figure 1:**
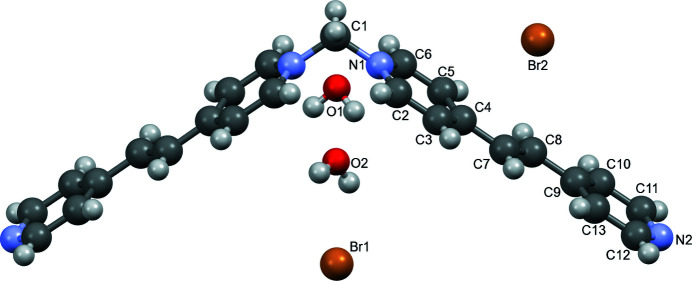
Ellipsoid (50%) representation of the title complex with disorder omitted for clarity.

Half of the cation is generated by the mirror plane (*x*, 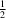 − *y*, *z*). The O1, O2, Br1, and C1 atoms are located on this mirror plane and the Br2 atom is on a twofold screw axis (−*x*, 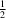 + *y*, −*z*). The pyrid­yl–vin­yl–pyridinium moiety (Fig. 1 ▸) is essentially planar with a 1.7 (3)° dihedral angle between the planes of the pyridinium (N1/C2–C6) and pyridyl (N2/C9–C13) rings. The N1—C1—N1(*x*, 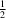 − *y*, *z*) angle is 110.9 (10)°, which is similar to the N—C—N angles of 111.1 (4) or 112.3 (4)° found in the bromide (Schuster *et al.* 2022 ▸) or PF_6_^−^ (Blanco *et al.*, 2007 ▸) salts, respectively, of the 1,1′-methyl­enebis-4,4′-bipyridinium cation. When two of the title cations are used in a supra­molecular cyclic compound with two Pd(ethyl­enedi­amine) moieties, the crystal structure had this same N—C—N angle remaining relatively unchanged at 109.1 (19) and 111.2 (11)° (Blanco *et al.*, 2009 ▸).

**Figure 2:**
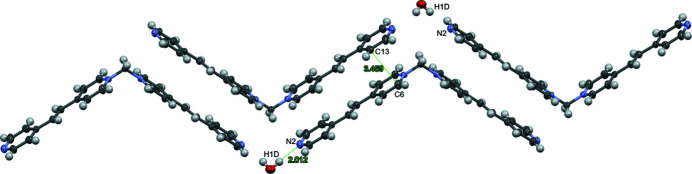
Zigzag ribbons composed of back-to-back chevron-shaped cations of the title complex. The distance between N2 and H1*D*( 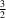 − *x*, 1 − *y*, 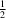 + *z*) is shown. Ellipsoids at 50% with disorder, bromide ions and some water mol­ecules omitted for clarity.

**Figure 3:**
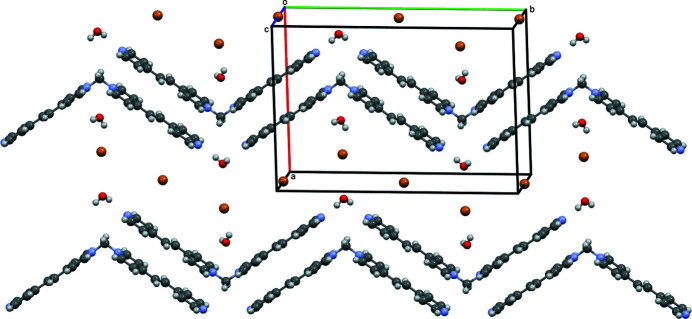
Ellipsoid (50%) representation of ribbons of cations with bromide ions (brown) and water mol­ecules positioned between them. Ellipsoids at 50% with disorder omitted for clarity.

In the extended structure, the chevron-shaped cations of the title compound arrange in back-to-back alternating directions to form a zigzag ribbon (Fig. 2 ▸) propagating along the [010] direction. Water mol­ecules are positioned to inter­act with the terminal pyridyl nitro­gen atom, N2, with an N2—H1*D*( 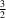 − *x*, 1 − *y*, 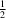 + *z*) distance of 2.01 Å (Table 1 ▸). The distance between back-to-back pyridinium and pyridyl rings [the closest distance between carbon atoms, C6 of the pyridinium and C13(1 − *x*, 1 − *y*, 1 − *z*) of a pyridyl ring, being 3.46 (1) Å (Fig. 2 ▸)] is suitable for π–π inter­actions (Sinnokrot *et al.*, 2002 ▸), which further consolidate these zigzag ribbons. Water molecules and bromide ions pack between the ribbons (Fig. 3 ▸). Other hydrogen-bonded zigzag ribbon structures are observed in 1,3-bis­[(tetra­hydro­furan-2-yl)meth­yl]thio­urea (Peña *et al.*, 2009 ▸) or 1-(4-bromo­phen­yl)-3-(4-eth­oxy­phen­yl)prop-2-en-1-one (Fun *et al.*, 2008 ▸).

## Synthesis and crystallization

The title compound was synthesized according to published procedures (Blanco *et al.*, 2009 ▸). Colorless plates were grown from liquid diffusion of tetra­hydro­furan into a di­methyl­formamide solution of the pyridinium bromide salt.

## Refinement

Crystal data, data collection and structure refinement details are summarized in Table 2 ▸. Disorder of the 4-[(*E*)-2-(pyridin-4-yl)ethen­yl]pyridinium moiety was refined using ‘PART 1’ and ‘PART 2’ with the ratio of occupancies at 47 and 53%. All our attempts to refine the structure to achieve equal occupancies led to a drastic worsening of *R*1 and *wR*2 values.

## Supplementary Material

Crystal structure: contains datablock(s) I. DOI: 10.1107/S2414314622005259/bx4021sup1.cif

Structure factors: contains datablock(s) I. DOI: 10.1107/S2414314622005259/bx4021Isup2.hkl

Supporting information file. DOI: 10.1107/S2414314622005259/bx4021Isup3.mol

Supporting information file. DOI: 10.1107/S2414314622005259/bx4021Isup4.cml

CCDC reference: 2173317

Additional supporting information:  crystallographic information; 3D view; checkCIF report

## Figures and Tables

**Table 1:** Hydrogen-bond geometry (Å, °)

*D*—H⋯*A*	*D*—H	H⋯*A*	*D*⋯*A*	*D*—H⋯*A*
O1—H1*C*⋯N2^i^	0.88	2.26	2.880 (11)	128
O1—H1*D*⋯N2^ii^	0.88	2.01	2.880 (11)	171

Symmetry codes: (i) 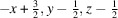 ; (ii) 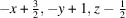 .

**Table 2:** Experimental details

Crystal data	
Chemical formula	C_25_H_22_N_4_^2+^·2Br^−^·2H_2_O
*M*_r_	574.32
Crystal system, space group	Orthorhombic, *P* *n* *m* *a*
Temperature (K)	220
*a*, *b*, *c* (Å)	15.4863 (2), 22.2936 (3), 7.2100 (1)
*V* (Å^3^)	2489.22 (6)
*Z*	4
Radiation type	Cu *K*α
μ (mm^−1^)	4.37
Crystal size (mm)	0.04 × 0.03 × 0.02

Data collection	
Diffractometer	XtaLAB Synergy, Dualflex, HyPix
Absorption correction	Multi-scan *CrysAlis PRO* (Rigaku OD, 2021 ▸)
*T*_min_, *T*_max_	0.671, 1.000
No. of measured, independent and observed [*I* > 2σ(*I*)] reflections	25704, 2780, 2439
*R*_int_	0.030
(sin θ/λ)_max_ (Å^−1^)	0.639

Refinement	
*R*[*F* ^2^ > 2σ(*F* ^2^)], *wR*(*F* ^2^), *S*	0.036, 0.104, 1.09
No. of reflections	2780
No. of parameters	244
No. of restraints	8
H-atom treatment	H atoms treated by a mixture of independent and constrained refinement
Δρ_max_, Δρ_min_ (e Å^−3^)	0.99, −0.86

Computer programs: *CrysAlis PRO* (Rigaku OD, 2021 ▸), *SHELXT2018/2* (Sheldrick, 2015*a* ▸), *SHELXL2018/3* (Sheldrick, 2015*b* ▸), *Mercury* (Macrae *et al.*, 2020 ▸), and *OLEX2* (Dolomanov *et al.*, 2009 ▸).

## full crystallographic data

### Crystal data

**Table:**

C_25_H_22_N_4_^2+^·2Br^−^·2H_2_O	*D*_x_ = 1.532 Mg m^−^^3^
*M**_r_* = 574.32	Cu *K*α radiation, λ = 1.54184 Å
Orthorhombic, *P**n**m**a*	Cell parameters from 14220 reflections
*a* = 15.4863 (2) Å	θ = 6.1–79.8°
*b* = 22.2936 (3) Å	µ = 4.37 mm^−^^1^
*c* = 7.2100 (1) Å	*T* = 220 K
*V* = 2489.22 (6) Å^3^	Plate, clear light colourless
*Z* = 4	0.04 × 0.03 × 0.02 mm
*F*(000) = 1160	

### Data collection

**Table:**

XtaLAB Synergy, Dualflex, HyPix diffractometer	2780 independent reflections
Radiation source: micro-focus sealed X-ray tube, PhotonJet (Cu) X-ray Source	2439 reflections with *I* > 2σ(*I*)
Mirror monochromator	*R*_int_ = 0.030
Detector resolution: 10.0000 pixels mm^-1^	θ_max_ = 80.3°, θ_min_ = 4.0°
ω scans	*h* = −17→19
Absorption correction: multi-scan CrysAlisPro (Rigaku OD, 2021)	*k* = −28→27
*T*_min_ = 0.671, *T*_max_ = 1.000	*l* = −9→9
25704 measured reflections	

### Refinement

**Table:**

Refinement on *F*^2^	8 restraints
Least-squares matrix: full	Hydrogen site location: mixed
*R*[*F*^2^ > 2σ(*F*^2^)] = 0.036	H atoms treated by a mixture of independent and constrained refinement
*wR*(*F*^2^) = 0.104	*w* = 1/[σ^2^(*F*_o_^2^) + (0.0464*P*)^2^ + 1.8748*P*] where *P* = (*F*_o_^2^ + 2*F*_c_^2^)/3
*S* = 1.09	(Δ/σ)_max_ < 0.001
2780 reflections	Δρ_max_ = 0.99 e Å^−^^3^
244 parameters	Δρ_min_ = −0.86 e Å^−^^3^

### Special details

**Table:**

Geometry. All esds (except the esd in the dihedral angle between two l.s. planes) are estimated using the full covariance matrix. The cell esds are taken into account individually in the estimation of esds in distances, angles and torsion angles; correlations between esds in cell parameters are only used when they are defined by crystal symmetry. An approximate (isotropic) treatment of cell esds is used for estimating esds involving l.s. planes.

### Fractional atomic coordinates and isotropic or equivalent isotropic displacement parameters (Å^2^)

**Table:**

	*x*	*y*	*z*	*U*_iso_*/*U*_eq_	Occ. (<1)
Br1	0.83013 (2)	0.250000	0.51496 (5)	0.05780 (16)	
Br2	0.500000	0.500000	0.000000	0.06787 (19)	
O1	0.59949 (18)	0.250000	−0.0276 (5)	0.0644 (5)	
H1C	0.628097	0.228712	0.053830	0.097*	0.5
H1D	0.637353	0.279127	−0.030494	0.097*	0.5
O2	0.61749 (17)	0.250000	0.5693 (5)	0.0644 (5)	
H2B	0.666046	0.234075	0.534382	0.097*	0.5
H2C	0.616243	0.229500	0.676421	0.097*	0.5
N1	0.4441 (7)	0.3047 (4)	0.4944 (11)	0.0280 (19)	0.471 (7)
C2	0.4622 (7)	0.3261 (4)	0.6635 (9)	0.0336 (16)	0.471 (7)
H2	0.441055	0.306491	0.769534	0.040*	0.471 (7)
C3	0.5120 (5)	0.3769 (3)	0.6814 (7)	0.0338 (14)	0.471 (7)
H3	0.523534	0.392117	0.800374	0.041*	0.471 (7)
C4	0.5451 (3)	0.4059 (2)	0.5289 (9)	0.0259 (12)	0.471 (7)
C5	0.5254 (4)	0.3821 (3)	0.3578 (7)	0.0355 (15)	0.471 (7)
H5	0.546674	0.400906	0.250362	0.043*	0.471 (7)
C6	0.4753 (6)	0.3316 (4)	0.3410 (8)	0.0375 (17)	0.471 (7)
H6	0.462840	0.315841	0.223042	0.045*	0.471 (7)
N2	0.7822 (9)	0.6508 (4)	0.5018 (14)	0.048 (3)	0.471 (7)
C11	0.7660 (7)	0.6282 (4)	0.3350 (11)	0.0430 (17)	0.471 (7)
H11	0.788849	0.647826	0.230565	0.052*	0.471 (7)
C10	0.7170 (5)	0.5769 (3)	0.3083 (8)	0.0383 (15)	0.471 (7)
H10	0.707421	0.562561	0.187380	0.046*	0.471 (7)
C9	0.6822 (3)	0.54692 (19)	0.4566 (10)	0.0301 (12)	0.471 (7)
C13	0.6980 (5)	0.5710 (3)	0.6290 (8)	0.0394 (16)	0.471 (7)
H13	0.674916	0.552711	0.735506	0.047*	0.471 (7)
C12	0.7480 (8)	0.6224 (4)	0.6452 (10)	0.050 (2)	0.471 (7)
H12	0.758017	0.637927	0.764548	0.060*	0.471 (7)
C1	0.39067 (17)	0.250000	0.4756 (4)	0.0281 (5)	
C7	0.5974 (3)	0.4592 (2)	0.5575 (7)	0.0328 (12)	0.471 (7)
H7	0.608461	0.470689	0.680642	0.039*	0.471 (7)
C8	0.6307 (3)	0.4929 (2)	0.4239 (6)	0.0321 (13)	0.471 (7)
H8	0.620576	0.481388	0.300419	0.038*	0.471 (7)
C8A	0.6252 (3)	0.4946 (2)	0.5660 (6)	0.0352 (12)	0.529 (7)
H8A	0.604322	0.485675	0.685211	0.042*	0.529 (7)
C7A	0.6033 (3)	0.4578 (2)	0.4290 (6)	0.0330 (11)	0.529 (7)
H7A	0.624114	0.466670	0.309581	0.040*	0.529 (7)
N1A	0.4453 (6)	0.3041 (3)	0.4673 (9)	0.0244 (15)	0.529 (7)
C2A	0.4791 (5)	0.3212 (3)	0.3033 (8)	0.0290 (12)	0.529 (7)
H2A	0.467537	0.298861	0.195459	0.035*	0.529 (7)
C3A	0.5305 (3)	0.3713 (2)	0.2945 (6)	0.0307 (12)	0.529 (7)
H3A	0.553479	0.383222	0.179661	0.037*	0.529 (7)
C4A	0.5489 (3)	0.40443 (19)	0.4506 (8)	0.0264 (11)	0.529 (7)
C5A	0.5131 (4)	0.3853 (3)	0.6147 (7)	0.0341 (13)	0.529 (7)
H5A	0.523793	0.407169	0.723830	0.041*	0.529 (7)
C6A	0.4621 (6)	0.3349 (3)	0.6226 (7)	0.0323 (13)	0.529 (7)
H6A	0.439049	0.322149	0.736540	0.039*	0.529 (7)
N2A	0.7859 (8)	0.6497 (4)	0.5226 (13)	0.052 (3)	0.529 (7)
C11A	0.7539 (7)	0.6311 (4)	0.6829 (10)	0.0468 (16)	0.529 (7)
H11A	0.767754	0.652790	0.790561	0.056*	0.529 (7)
C10A	0.7011 (4)	0.5811 (3)	0.7003 (7)	0.0401 (14)	0.529 (7)
H10A	0.680077	0.569719	0.817495	0.048*	0.529 (7)
C9A	0.6796 (3)	0.54816 (18)	0.5456 (9)	0.0325 (11)	0.529 (7)
C13A	0.7118 (4)	0.5682 (3)	0.3798 (8)	0.0442 (15)	0.529 (7)
H13A	0.698343	0.547622	0.269787	0.053*	0.529 (7)
C12A	0.7641 (7)	0.6187 (4)	0.3728 (10)	0.054 (2)	0.529 (7)
H12A	0.785018	0.631399	0.256891	0.065*	0.529 (7)
H1A	0.359 (2)	0.250000	0.584 (4)	0.025 (7)*	
H1B	0.363 (2)	0.250000	0.372 (5)	0.036 (9)*	

### Atomic displacement parameters (Å^2^)

**Table:**

	*U*^11^	*U*^22^	*U*^33^	*U*^12^	*U*^13^	*U*^23^
Br1	0.0341 (2)	0.1080 (4)	0.03128 (19)	0.000	0.00029 (12)	0.000
Br2	0.1255 (5)	0.0507 (2)	0.0274 (2)	0.0236 (2)	0.00356 (18)	0.00004 (13)
O1	0.0401 (9)	0.0554 (10)	0.0978 (16)	0.000	−0.0060 (10)	0.000
O2	0.0401 (9)	0.0554 (10)	0.0978 (16)	0.000	−0.0060 (10)	0.000
N1	0.023 (3)	0.025 (4)	0.036 (3)	0.001 (3)	0.004 (2)	0.007 (3)
C2	0.045 (3)	0.030 (3)	0.025 (3)	−0.006 (3)	0.007 (3)	−0.003 (2)
C3	0.046 (3)	0.033 (3)	0.022 (3)	−0.006 (2)	0.003 (3)	−0.008 (3)
C4	0.029 (2)	0.023 (2)	0.026 (3)	0.0000 (16)	0.002 (2)	−0.004 (3)
C5	0.048 (3)	0.033 (3)	0.025 (4)	−0.002 (2)	0.008 (3)	0.008 (3)
C6	0.051 (4)	0.036 (3)	0.026 (3)	−0.003 (3)	−0.010 (3)	−0.004 (3)
N2	0.039 (6)	0.025 (6)	0.078 (6)	0.000 (5)	0.003 (5)	0.007 (5)
C11	0.042 (3)	0.028 (3)	0.060 (4)	−0.005 (2)	0.005 (3)	0.006 (3)
C10	0.040 (3)	0.035 (3)	0.040 (4)	−0.002 (2)	0.004 (3)	−0.001 (3)
C9	0.028 (2)	0.023 (2)	0.039 (4)	0.0008 (16)	0.001 (3)	0.001 (3)
C13	0.040 (3)	0.036 (4)	0.042 (4)	−0.004 (2)	0.000 (3)	0.001 (3)
C12	0.052 (4)	0.032 (3)	0.065 (5)	−0.001 (3)	−0.014 (4)	−0.011 (3)
C1	0.0243 (12)	0.0240 (11)	0.0360 (14)	0.000	−0.0007 (11)	0.000
C7	0.033 (2)	0.027 (3)	0.038 (3)	−0.0030 (19)	0.0006 (18)	−0.0038 (17)
C8	0.032 (2)	0.028 (3)	0.035 (3)	−0.0021 (19)	−0.0027 (17)	−0.0009 (16)
C8A	0.0346 (19)	0.032 (2)	0.039 (3)	−0.0033 (18)	0.0025 (16)	0.0023 (16)
C7A	0.0326 (19)	0.029 (2)	0.037 (2)	−0.0024 (17)	0.0024 (15)	0.0007 (16)
N1A	0.026 (3)	0.022 (3)	0.026 (2)	0.000 (2)	−0.004 (2)	−0.004 (2)
C2A	0.034 (2)	0.032 (3)	0.022 (2)	−0.008 (2)	0.0000 (18)	0.0004 (19)
C3A	0.032 (2)	0.034 (2)	0.026 (3)	−0.0048 (18)	0.004 (2)	0.003 (2)
C4A	0.0276 (18)	0.027 (2)	0.024 (3)	0.0010 (14)	−0.001 (2)	−0.008 (2)
C5A	0.047 (3)	0.032 (3)	0.024 (3)	−0.002 (2)	0.000 (3)	−0.008 (3)
C6A	0.039 (3)	0.034 (3)	0.023 (3)	0.001 (2)	0.006 (2)	−0.002 (2)
N2A	0.041 (5)	0.033 (6)	0.081 (5)	−0.010 (5)	−0.001 (5)	−0.007 (5)
C11A	0.045 (3)	0.035 (3)	0.061 (4)	−0.006 (2)	−0.007 (3)	−0.007 (3)
C10A	0.042 (2)	0.034 (3)	0.044 (3)	−0.001 (2)	−0.006 (3)	0.000 (3)
C9A	0.0290 (19)	0.028 (2)	0.041 (3)	0.0021 (16)	−0.001 (2)	−0.003 (3)
C13A	0.053 (3)	0.037 (3)	0.042 (4)	−0.007 (2)	0.005 (3)	−0.005 (3)
C12A	0.058 (4)	0.036 (3)	0.068 (5)	−0.005 (3)	0.017 (4)	0.003 (3)

### Geometric parameters (Å, º)

**Table:**

O1—H1C	0.8753	C1—N1A^i^	1.474 (4)
O1—H1C^i^	0.88 (6)	C1—H1A	0.92 (3)
O1—H1D	0.8752	C1—H1B	0.86 (4)
O1—H1D^i^	0.88 (8)	C7—H7	0.9400
O2—H2B	0.8688	C7—C8	1.326 (7)
O2—H2B^i^	0.87 (5)	C8—H8	0.9400
O2—H2C	0.8975	C8A—H8A	0.9400
O2—H2C^i^	0.90 (6)	C8A—C7A	1.328 (6)
N1—C2	1.3385	C8A—C9A	1.468 (6)
N1—C6	1.3481	C7A—H7A	0.9400
N1—C1	1.480 (5)	C7A—C4A	1.467 (7)
C2—H2	0.9400	N1A—C2A	1.3482
C2—C3	1.3755	N1A—C6A	1.3387
C3—H3	0.9400	C2A—H2A	0.9400
C3—C4	1.3742	C2A—C3A	1.3735
C4—C5	1.3759	C3A—H3A	0.9400
C4—C7	1.453 (7)	C3A—C4A	1.3761
C5—H5	0.9400	C4A—C5A	1.3737
C5—C6	1.3734	C5A—H5A	0.9400
C6—H6	0.9400	C5A—C6A	1.3753
N2—C11	1.3275	C6A—H6A	0.9400
N2—C12	1.3235	N2A—C11A	1.3241
C11—H11	0.9400	N2A—C12A	1.3271
C11—C10	1.3868	C11A—H11A	0.9400
C10—H10	0.9400	C11A—C10A	1.3883
C10—C9	1.3715	C10A—H10A	0.9400
C9—C13	1.3756	C10A—C9A	1.3758
C9—C8	1.464 (7)	C9A—C13A	1.3709
C13—H13	0.9400	C13A—H13A	0.9400
C13—C12	1.3883	C13A—C12A	1.3864
C12—H12	0.9400	C12A—H12A	0.9400
C1—N1A	1.474 (4)		
			
H1C—O1—H1C^i^	65.7	N1A—C1—H1A	110.0 (10)
H1C^i^—O1—H1D^i^	94.5	N1A^i^—C1—H1A	110.0 (10)
H1C—O1—H1D^i^	43.5	N1A^i^—C1—H1B	104.5 (13)
H1C—O1—H1D	94.5	N1A—C1—H1B	104.5 (13)
H1D—O1—H1C^i^	43.5	H1A—C1—H1B	118 (3)
H1D—O1—H1D^i^	95.8	C4—C7—H7	117.4
H2B—O2—H2B^i^	48.2	C8—C7—C4	125.3 (6)
H2B^i^—O2—H2C^i^	93.4	C8—C7—H7	117.4
H2B—O2—H2C^i^	118.4	C9—C8—H8	117.9
H2B—O2—H2C	93.4	C7—C8—C9	124.2 (5)
H2C—O2—H2B^i^	118.4	C7—C8—H8	117.9
H2C—O2—H2C^i^	61.2	C7A—C8A—H8A	117.5
C2—N1—C6	120.9	C7A—C8A—C9A	125.1 (5)
C2—N1—C1	119.6 (5)	C9A—C8A—H8A	117.5
C6—N1—C1	119.4 (5)	C8A—C7A—H7A	117.7
N1—C2—H2	120.1	C8A—C7A—C4A	124.7 (5)
N1—C2—C3	119.8	C4A—C7A—H7A	117.7
C3—C2—H2	120.1	C2A—N1A—C1	119.3 (4)
C2—C3—H3	119.3	C6A—N1A—C1	119.8 (4)
C4—C3—C2	121.4	C6A—N1A—C2A	120.9
C4—C3—H3	119.3	N1A—C2A—H2A	120.2
C3—C4—C5	117.0	N1A—C2A—C3A	119.7
C3—C4—C7	118.6 (4)	C3A—C2A—H2A	120.2
C5—C4—C7	124.4 (4)	C2A—C3A—H3A	119.4
C4—C5—H5	119.4	C2A—C3A—C4A	121.3
C6—C5—C4	121.3	C4A—C3A—H3A	119.4
C6—C5—H5	119.4	C3A—C4A—C7A	117.9 (4)
N1—C6—C5	119.7	C5A—C4A—C7A	125.1 (4)
N1—C6—H6	120.2	C5A—C4A—C3A	117.0
C5—C6—H6	120.2	C4A—C5A—H5A	119.3
C12—N2—C11	116.8	C4A—C5A—C6A	121.4
N2—C11—H11	118.6	C6A—C5A—H5A	119.3
N2—C11—C10	122.9	N1A—C6A—C5A	119.8
C10—C11—H11	118.6	N1A—C6A—H6A	120.1
C11—C10—H10	119.7	C5A—C6A—H6A	120.1
C9—C10—C11	120.6	C11A—N2A—C12A	116.8
C9—C10—H10	119.7	N2A—C11A—H11A	118.2
C10—C9—C13	116.4	N2A—C11A—C10A	123.5
C10—C9—C8	119.3 (5)	C10A—C11A—H11A	118.2
C13—C9—C8	124.3 (5)	C11A—C10A—H10A	120.1
C9—C13—H13	120.1	C9A—C10A—C11A	119.8
C9—C13—C12	119.8	C9A—C10A—H10A	120.1
C12—C13—H13	120.1	C10A—C9A—C8A	119.4 (4)
N2—C12—C13	123.5	C13A—C9A—C8A	124.2 (4)
N2—C12—H12	118.2	C13A—C9A—C10A	116.4
C13—C12—H12	118.2	C9A—C13A—H13A	119.7
N1^i^—C1—N1	110.9 (10)	C9A—C13A—C12A	120.6
N1—C1—H1A	102.9 (11)	C12A—C13A—H13A	119.7
N1^i^—C1—H1A	102.9 (11)	N2A—C12A—C13A	122.8
N1—C1—H1B	110.9 (11)	N2A—C12A—H12A	118.6
N1^i^—C1—H1B	110.9 (11)	C13A—C12A—H12A	118.6
N1A—C1—N1A^i^	109.8 (9)		
			
N1—C2—C3—C4	−1.2	C7—C4—C5—C6	−179.8 (6)
C2—N1—C6—C5	−0.9	C8—C9—C13—C12	179.8 (6)
C2—N1—C1—N1^i^	−84.1 (7)	C8A—C7A—C4A—C3A	178.8 (4)
C2—C3—C4—C5	0.7	C8A—C7A—C4A—C5A	0.2 (6)
C2—C3—C4—C7	−179.8 (6)	C8A—C9A—C13A—C12A	−178.8 (6)
C3—C4—C5—C6	−0.3	C7A—C8A—C9A—C10A	−176.9 (4)
C3—C4—C7—C8	−177.3 (5)	C7A—C8A—C9A—C13A	2.7 (7)
C4—C5—C6—N1	0.4	C7A—C4A—C5A—C6A	179.4 (5)
C4—C7—C8—C9	179.1 (4)	N1A^i^—C1—N1A—C2A	76.4 (7)
C5—C4—C7—C8	2.2 (7)	N1A^i^—C1—N1A—C6A	−102.3 (5)
C6—N1—C2—C3	1.3	N1A—C2A—C3A—C4A	0.5
C6—N1—C1—N1^i^	94.7 (7)	C2A—N1A—C6A—C5A	1.2
N2—C11—C10—C9	0.2	C2A—C3A—C4A—C7A	−179.2 (5)
C11—N2—C12—C13	0.7	C2A—C3A—C4A—C5A	−0.5
C11—C10—C9—C13	0.8	C3A—C4A—C5A—C6A	0.8
C11—C10—C9—C8	180.0 (6)	C4A—C5A—C6A—N1A	−1.2
C10—C9—C13—C12	−1.1	C6A—N1A—C2A—C3A	−0.9
C10—C9—C8—C7	177.0 (5)	N2A—C11A—C10A—C9A	0.0
C9—C13—C12—N2	0.3	C11A—N2A—C12A—C13A	−1.1
C13—C9—C8—C7	−3.9 (7)	C11A—C10A—C9A—C8A	178.7 (6)
C12—N2—C11—C10	−1.0	C11A—C10A—C9A—C13A	−1.0
C1—N1—C2—C3	180.0 (10)	C10A—C9A—C13A—C12A	0.8
C1—N1—C6—C5	−179.6 (9)	C9A—C8A—C7A—C4A	−180.0 (4)
C1—N1A—C2A—C3A	−179.6 (8)	C9A—C13A—C12A—N2A	0.2
C1—N1A—C6A—C5A	179.9 (8)	C12A—N2A—C11A—C10A	1.0

Symmetry code: (i) *x*, −*y*+1/2, *z*.

### Hydrogen-bond geometry (Å, º)

**Table:**

*D*—H···*A*	*D*—H	H···*A*	*D*···*A*	*D*—H···*A*
O1—H1*C*···N2^ii^	0.88	2.26	2.880 (11)	128
O1—H1*D*···N2^iii^	0.88	2.01	2.880 (11)	171

Symmetry codes: (ii) −*x*+3/2, *y*−1/2, *z*−1/2; (iii) −*x*+3/2, −*y*+1, *z*−1/2.
